# Termitophily Documented in Earwigs (Dermaptera)

**DOI:** 10.3390/biology10121243

**Published:** 2021-11-28

**Authors:** Petr Kočárek, Rodzay Abdul Wahab

**Affiliations:** 1 Department of Biology and Ecology, Faculty of Science, University of Ostrava, Chittussiho 10, CZ-710 00 Ostrava, Czech Republic; 2 Institute for Biodiversity and Environmental Research, Universiti Brunei Darussalam, Jalan Tungku Link Gadong BE 1410, Brunei; rodzay.wahab@ubd.edu.bn

**Keywords:** Spongiphoridae, Labiinae, Rhinotermitidae, Schedorhinotermes, termitophile, symbiosis, Borneo

## Abstract

**Simple Summary:**

In the current report, we describe a new earwig species, *Spirolabia kaja* Kočárek, sp. nov. found in an association with the wood-boring termite in a dipterocarp rain forest in Borneo. To evaluate the level of association with termites we documented termite–earwig interactions in the laboratory. We found that earwigs and termites mutually communicate by antennation, and we observed no form of aggressive behavior. The earwigs responded to the proximity of an experimentally irritated termite soldier by the feigning of death (thanatosis), which seems to be a defensive reaction. The occurrence of earwig adults together with the nymphs in the galleries termite strongly suggests that the earwig reproduces inside the termite colony. *Spirolabia kaja* Kočárek, sp. nov. is the first earwig species for which termitophily has been demonstrated.

**Abstract:**

Based on behavioral observations, we report termitophily by the earwig *Spirolabia kaja* Kočárek, sp. nov. (Spongiphoridae: Labiinae). The new species was found in association with the wood-boring termite *Schedorhinotermes sarawakensis* (Holmgren, 1913) in a dipterocarp rain forest in Borneo; in addition to being observed in the galleries, termite–earwig interactions were subsequently documented in the laboratory. We found that earwigs and termites communicate by antennation, and we observed no form of targeted mutual or unilateral aggressive behavior. The earwigs responded to the proximity of an experimentally irritated termite soldier by conflict-avoidance behavior based on thanatosis, which seems to be a defensive reaction that may reduce the chance of being attacked by an irritated termite. Based on the analysis of gastrointestinal tract contents, we conclude that *S. kaja* sp. nov. is an omnivorous species that feeds mainly on plant tissues and fungi but occasionally on arthropod remains. The occurrence of *S. kaja* sp. nov. adults together with the nymphs (2nd to 4th instars) in the galleries of *S. sarawakensis* strongly suggests that the earwig can reproduce inside the termite colony. *Spirolabia kaja* Kočárek, sp. nov. is the first earwig species for which termitophily has been demonstrated.

## 1. Introduction

Social insect nests, including termite mounds, provide a rich microhabitat, often containing abundant and long-lasting resources such as brood, retrieved or cultivated food, and nutrient-rich refuse [1,2]. Termite nests are therefore attractive habitats for many associated species that benefit from the stable environment, protection against enemies, and the availability of food regularly supplied by the termites [3]. Such associated species are referred to as termitophiles, which include persecuted guests and tolerated guests, as well as highly specialized termitophiles that have evolved chemical, morphological, and behavioral adaptations that deceive the host and thereby reduce the probability of attack by the host [2,3,4,5,6]. An animal that lives in a termite nest for at least one complete stage of its life cycle can be considered a termitophile [5].

The ability to function as a termitophile has evolved in many terrestrial insect lineages, including the Coleoptera, Diptera, Hymenoptera, Lepidoptera, Heteroptera, and Blattodea, among others [5,6,7,8]. Nevertheless, termitophily is heavily skewed to particular groups of arthropods that appear preadapted to a shift toward this peculiar life history. These preadapted groups include small scavenging or predatory arthropods with chemical, physical or behavioral defenses [2]. 

Some groups of earwigs (Dermaptera) seem to be good candidates for termitophily due to their small size, scavenging feeding habit, visual and size similarity with termites, ability to move quickly, and ability to produce defensive chemicals [9]. Many free-living earwigs share the same habitats as termites, such as forest litter or rotting wood. The evolutionary transition from a free-living lifestyle to termitophily can therefore be expected, and it is surprising that no termitophilic species of earwig has been recorded to date. In an extensive study of the literature on earwigs, we found only one mention of potential termitophily, and that mention concerned a newly described species of Spongiphoridae, *Paralabellula termitophila* (Brindle, 1970). A specimen of that species was found in the nest of a nasute termite on Solomon Islands. The nature of its association with termites, however, was unknown at the time of its description [10] and has since remained uninvestigated, although it was reasonable to suspect some form of termitophily. 

In the current report, we describe a new earwig species found in an association with a termite in the genus *Schedorhinotermes* Silvestri, 1909 (Rhinotermitidae). The new species was discovered during an ecological study conducted in 2014 and 2015 in a mixed lowland dipterocarp forest in Brunei Darussalam on the island of Borneo. We document behavioral interactions between earwigs and termites and make inferences on the feeding habits of the earwig based on gastrointestinal tract analysis.

## 2. Materials and Methods

### 2.1. Material Collection and Identification

The research was conducted in the lowland mixed dipterocarp forest in Ulu Temburong, Brunei Darussalam. The earwigs were found in the termite galleries in dead rotting logs (60–80 cm in diameter) in the Sungai Esu stream valley (GPS: 04°32′14.1″ N, 115°9′47.1″ E) on 14 January 2014 and on 17 February 2015, each year in only 1 termite colony. Earwigs were observed to move together with termite workers accompanied by minor and major soldiers. We first observed the earwigs in 2014, when only earwigs (not termites) were collected and stored in ethanol for taxonomic study. In 2015, the earwigs were found again in the termite galleries on a neighboring log and were taken alive to the laboratory together with the termites from the same gallery for behavioral observations (see below). After laboratory observations were completed, the earwigs and termites were stored in 75% ethanol for further studies and DNA isolation.

The earwigs, which were identified according to Steinmann [11] and Sakai [12], were found to be an undescribed species of the genus *Spirolabia*. The termites were found to belong to the genus *Schedorhinotermes* by David T. Jones (London, UK) according to detailed photographs; they were later identified to the species level according to Maiti [13] by the first author of this study.

### 2.2. Taxonomy

The studied specimens of *Spirolabia kaja* sp. nov. were removed from ethanol and dry-mounted, examined, and photographed with an Olympus SZ61 stereomicroscope equipped with a CANON D1000 camera. Genitalia were studied and photographed with an Olympus CX41 microscope equipped with a CANON D1000 camera. Microphotographs of 10 to 40 focal layers of the same specimen were combined with Quick Photo Camera 2.3 software and finally processed with Adobe Photoshop CS6 Extended (version 13). The type specimen was mounted on a label, and dissected body parts (penultimate sternites) were mounted with methylcellulose glue on the same board as the specimen. A genital armature was mounted in dimethyl hydantoin formaldehyde resin (DMHF, a water-soluble mounting medium) on the same label as the specimen. The nomenclature and terminology used for morphological characteristics follows that of Steinmann [11]; the terminology used to describe the terminalia and genitalia follows that of Kamimura [14]. The type specimens were deposited in the National Museum, Praha, Czech Republic (NMPC); in the Institute for Biodiversity and Environmental Research, Universiti Brunei Darussalam, Brunei Darussalam (UBDC); and in the collection of P. Kočárek, University of Ostrava, Czech Republic (PKCO).

### 2.3. Behavioral Observations

Termite–earwig interactions were observed in a plastic Petri dish arena (10 cm in diameter). Dry leaves of a Dipterocarpaceae tree were glued to the bottom of the arena so that the insects could not climb under them and were constantly observable. Wood from the termite gallery was sparsely scattered on the surface of the leaves. The observations were conducted in the laboratory of the Kuala Belalong Field Study Centre in Ulu Temburong National Park under ambient temperature and humidity conditions and under indirect diffused light (24–25 °C and 90–95% relative humidity). The arena was open from above during the observations. Earwigs and termites were separately transported from the collection locality in plastic tubes and were placed together in the arena at 0.5 h before the start of observation. The observations were made for 2 successive hours that began 2.5 h after insect collection on 17 January 2015 (collection time was 15:30, observation time 18:00–20:00). The observed specimens in the arena included concurrently 5 major soldiers, 5 minor soldiers, 5 workers, and 5 earwigs (1 male, 2 females, and 2 nymphs). The number of termites added to the arena was adapted to the size of the arena in order to provide sufficient space for the earwigs to move out of the reach of the termites. Interactions were observed without researcher intervention except when we wanted to monitor the reaction of earwigs to the irritated termite soldiers. In the latter case, the soldiers were mechanically irritated with tweezers, and the reactions of freely passing earwigs were monitored. Observations with irritated soldiers were performed in the last 15 min of the 2-h observation period; no random earwig–termite interactions had been observed in the previous 1.75 h. Interactions between termites and earwigs were recorded and documented with a Canon EOS 700D equipped with a Canon EF-S 60 Macro Lens and attached ring flesh MR-14EX II with diffuser.

### 2.4. Gastrointestinal Tract Analysis

The composition of the earwig diet was determined by analysing the gastrointestinal tract contents as described by Kocarek et al. [15], and the goal was to assess the possibility of predation on termites. The collected insects were immediately stored in ethanol (75%) to stop the digestion of food within the alimentary tract. In the dissection of these specimens, the sternites of the thorax and abdomen were cut using slender forceps, and the oesophagus, crop, proventriculus, and midgut were removed. The gastrointestinal tracts of three specimens of *Spirolabia kaja* sp. nov. were removed; one was from a male, and two were from females. Permanent microscopic preparations of the alimentary tract contents were made with Hoyer’s solution [16]. The slides were examined for visually identifiable fragments of food (animal, plant, and fungal), and these were documented using an Olympus CX41 microscope and a Canon EOS 1100D camera. The proportions of different kinds of foods were not determined due to the limited quantity of material and the predominance of indistinguishable small fragments.

### 2.5. Molecular Methods 

Cytochrome c oxidase (COI) barcode of *Spirolabia kaja* sp. nov. was determined and placed on GenBank to fix the species identity for the purpose of further taxonomic studies. Total genomic DNA was extracted from abdominal muscle tissue or legs of *S. kaja* adults using the QIAamp DNA Micro Kit (QIAGEN, Hilden, Germany) following the manufacturer’s protocol. Cytochrome c oxidase was amplified by polymerase chain reaction (PCR) with the universal pair of primers LCO1490/HCO2198 [17]. Standard PCR was conducted in 20-μL reaction volumes containing 1 μL of DNA template, 0.4 μM of each primer, 5× MyTaq Red PCR buffer, 0.5 U/μL of MyTaq™ Red DNA polymerase (Bioline Reagents, London, UK), 0.6 mg/mL bovine serum albumin (New England Biolabs Inc., Ipswich, MA, USA), and distilled water. The PCR cycling profile was as follows: 2 min at 94 °C for initial denaturation; followed by 35 cycles of 15 s at 94 °C, 15 s at 51 °C, and 15 s at 72 °C; and a final extension at 72 °C for 6 min.

The amplified DNA was purified using the GenElute PCR Clean-up Kit (Sigma-Aldrich, St. Louis, MO, USA) following the manufacturer’s protocol. Sanger sequencing reactions were performed using an ABI3730XL DNA sequencer by Macrogen Europe (Amsterdam, The Netherlands). The chromatograms were visually checked and manually edited where appropriate using Chromas v2.6.4 software (Technelysium, Brisbane, Australia). 

## 3. Results

### 3.1. Taxonomy

Order Dermaptera De Geer, 1773

Suborder Neodermaptera Engel, 2003

Infraorder Epidermaptera Engel, 2003

Superfamily Forficuloidea Latreille, 1810

Family Spongiphoridae Verhoeff, 1902

Subfamily Labiinae Burr, 1909

Genus *Spirolabia* Steinmann, 1987

**Figure 1:**
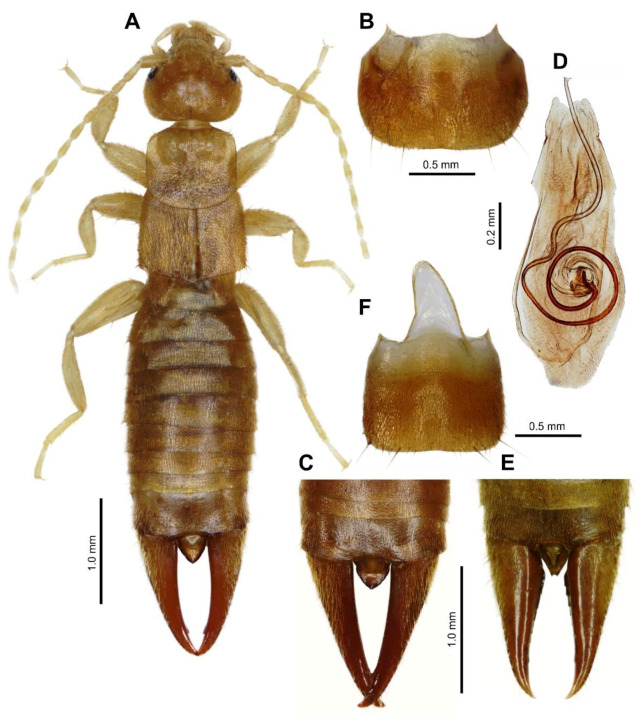
*Spirolabia kaja* Kočárek, sp. nov. (**A**)—habitus of holotype male from dorsal view; (**B**)—penultimate sternite of holotype male, ventral view; (**C**)—dorsal view on abdomen tip in paratype male; (**D**)—holotype male genital armature, dorsal view; (**E**)—dorsal view on abdomen tip in paratype female; (**F**)—penultimate sternite of paratype female.

**Figure 2:**
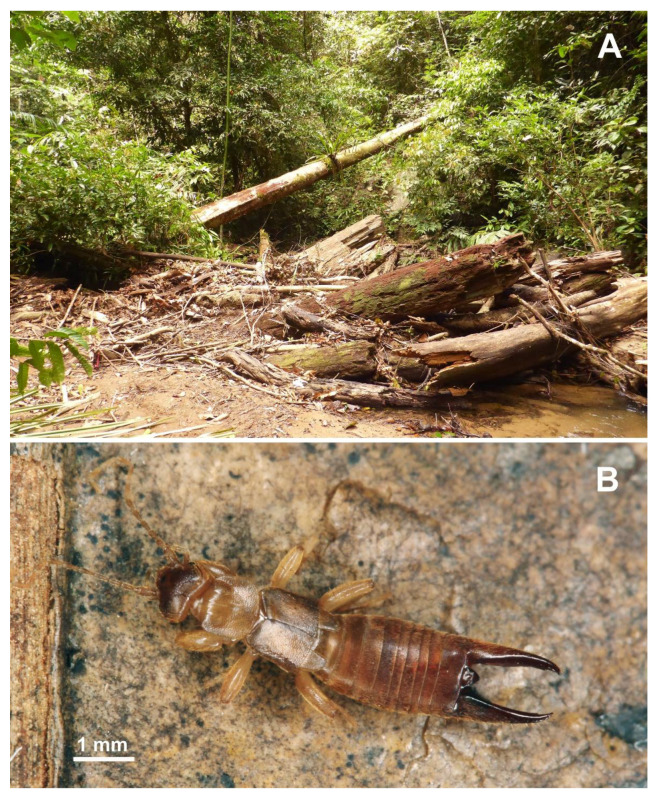
*Spirolabia kaja* Kočárek, sp. nov. (**A**)—type locality in the Sungai Esu stream valley in Ulu Temburong National Park, Brunei Darussalam; (**B**)—a living male of *Spirolabia kaja* sp. nov.

Species *Spirolabia kaja* Kočárek, sp. nov.

urn:lsid:zoobank.org:act:522E264A-D5A7-4BCE-BE42-F4D6CAB45849; Figure 1 and Figure 2.

Type locality. Brunei Darussalam: Ulu Temburong NP, Sungai Esu stream.

Material examined. Holotype male, labelled ‘Brunei Darussalam, 9.i.2014, Ulu Temburong NP, Sungai Esu stream, 150 m a.s.l., GPS: 04°32′14.1″ N, 115°9′47.1″ E, P. Kočárek leg.’ (NMPC), in termite galleries together with *Schedorhinotermes sarawakensis* (Holmgren, 1913) in rotting log [GenBank: OL505862; isolate number: 26-DE]. Paratypes: 1 female same data as for holotype (UBDC); 1 male, 1 female [GenBank: OL505863; isolate number: 27-DE] and 2 nymphs of 2nd/3rd instar: ‘Brunei Darussalam, 17.ii.2015, Ulu Temburong NP, Sungai Esu stream, 150 m a.s.l., GPS: 04°32′14.1″ N, 115°9′47.1″ E, P. Kočárek leg.’ (PKCO), in termite galleries together with *Schedorhinotermes sarawakensis* in rotting log; non-type material: 2 nymphs of 2nd/3rd instar, 1 nymph of 4th instar: ‘Brunei Darussalam, 17.ii.2015, Ulu Temburong NP, Sungai Esu stream, 150 m a.s.l., GPS: 04°32′14.1″ N, 115°9′47.1″ E, P. Kočárek leg.’ (PKCO). 

Description. General color ochre, legs and antennae lighter, forceps slightly darker; body covered by short pubescence. Total length 5.9–6.4 mm, length of forceps 1.3–1.5 mm.

Male. Head (Figure 1A) as long as broad; frons moderately convex, slightly punctate; sutures shallow but visible. Eyes small, not protruding, two times shorter than the postocular area, and about half the length of the basal antenna segment. Antennae 14-segmented; basal segment (scapus) stout, gently narrowed at base, shorter than the distance between antenna bases; 2nd segment (pedicellus) short, broader than long; 3rd segment longer than 1st and as long as 4th segment.

Pronotum (Figure 1A) transverse, slightly broader than long, lateral sides parallel, posterior margin broadly rounded, median sulcus faintly marked; prozona gently raised and metazona flat. Prosternum longer than broad, slightly narrowed posteriorly; mesosternum approximately as long as broad, hind margin convex and metasternum transverse, projecting as a narrow lobe between hind coxae with hind margin truncate. Tegmina well developed, broader than long (measured both together), punctate and densely covered by short setae; wings present but reduced, project from postero-lateral margins of tegmina as a pair of small non-contiguous triangles each approximately one-third the length of a tegmin. Legs typical for genus, covered by short setae, hind tarsi with 1st segment slightly longer than the combined length of the 2nd and 3rd segments. Claws simple, thin, symmetrical; arolium absent.

Abdomen (Figure 1A–C) slightly expanded at base, dorsoventrally flattened, punctate, densely covered by short setae; lateral tubercles (glandular folds) only on 4th tergite; abdominal tergite lateral without carina. Penultimate (9th) sternite (Figure 1B) transverse, broader than long, hind margin broadly rounded, with weak emargination in middle. Ultimate tergite (10th) transverse (Figure 1A,C), broader than long, punctulate, sloping backwards, median sulcus not marked; the area in middle posteriorly depressed, hind margin slightly emarginated in middle. Pygidium robust (Figure 1A,C), triangularly pointed, longer than broad. Forceps symmetrical (Figure 1A,C), with branches subremote at base, cylindrical, not depressed, tapering; tips pointed, both branches with minute triangular tooth near apex internally.

Male genitalia (Figure 1D) with small and short parameres rounded at apex; penis lobe broad, apex excised in middle; virga long, strongly sclerotized and coiled basally.

Female. Agrees with male in all characters except female pygidium is narrower (Figure 1E), excised in the middle distally; penultimate (9th) sternite (Figure 1F) is more quadratic; forceps are shorter and more robust, triangular tooth near apex is only faintly indicated (Figure 1E).

Differential diagnosis. *Spirolabia kaja* sp. nov. differs from other species of *Spirolabia* in characteristic male genitalia (Figure 1D) with spirally coiled virga with 1.5 revolutions, very short parameres and medially excised apex of genital lobe. Spirally coiled virga is a diagnostic character of *Spirolabia*, but in all other species the spiral is composed of 0.5–1.0 revolutions. Excised apex of genital lobe is a unique character in *Spirolabia*, but genitalia of not all known species are described. *S. kaja* sp. nov. has reduced wings with scales projecting from the postero-lateral margins of tegmina (Figure 1A and Figure 2B). Such arrangement of wings is reported in *S. kermadecensis* (Giles, 1973), but the later species has different genitalia, different shape of male forceps and pygidium [10,11]. Only one species of *Spirolabia* has been previously reported from Borneo: *S. pillicornis* (Motschulsky, 1863) [11,18,19,20]. 

Molecular identification. We obtained partial COI sequences (678 bp) from 2 specimens of *Spirolabia kaja* Kočárek, sp. nov. as DNA barcode for the purpose of molecular identification of the species and we deposited both in GenBank under accession numbers OL505862 (isolate number: 26-DE) and OL505863 (isolate number: 27-DE).

Etymology. The species name is dedicated to the first author’s older son, who likes earwigs. The name is derived from the domestic form of the name Karel (noun is in apposition). 

Habitat and bionomy. Lowland dipterocarp forest (Figure 2A). Termitophile species associated with wood boring *Schedorhinotermes sarawakensis*.

Distribution. Borneo, Brunei Darussalam: Ulu Temburong NP.

Taxonomic comment. Steinmann [11,21] erected three genera for species previously classified in Labia Leach, 1815: *Circolabia* Steinmann, 1987; *Spirolabia* Steinmann, 1987; and *Paralabella* Steinmann, 1990. These three genera differ from Labia by not excised parameres on male genitalia, and the differences among them were based on characters of the male genitalia: the shape of virga (coiled or not) and presence/absence of basal plate. Srivastava [18] found overlap between the above-mentioned diagnostic characters in all three genera and therefore synonymized *Spirolabia* and *Paralabella* (later synonymized with *Paralabellula* Kevan, 1997 [22]) with the oldest available name *Circolabia*. Kamimura et al. [23] considered the differences in the shapes of the spermathecae of *Paralabellula* and *Spirolabia* and confirmed the validity of the genera erected by Steinmann [1].

### 3.2. Earwig–Termite Interactions

**Figure 3:**
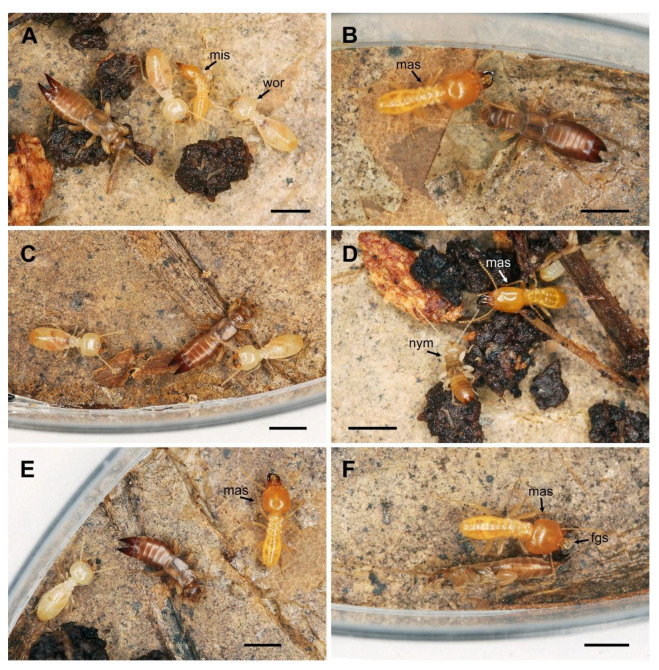
Interactions between *Spirolabia kaja* Kočárek, sp. nov. and *Schedorhinotermes sarawakensis* (Holmgren, 1913) in experimental arena under laboratory conditions. (**A**)—An *S. kaja* sp. nov. female in the presence of workers (wor) and a minor soldier (mis) of *Schedorhinotermes sarawakensis*; (**B**)—antennation between a major soldier (mas) of *S. sarawakensis* and a female of *S. kaja*; (**C**)—Avoidance of a termite worker by an *S. kaja* female after mutual antennation; (**D**)—mutual antennation between a major soldier of *S. sarawakensis* (mis) and a nymph (nym) of *S. kaja*; (**E**)—female of *S. kaja* between a worker and a major soldier (mas) of *Schedorhinotermes sarawakensis*; (**F**)—male of *S. kaja* exhibiting thanatosis after contact with an irritated major soldier (mas) with a secretion (fgs) from its frontal gland. Scale bar = 2 mm.

During contact of an adult *Spirolabia kaja* sp. nov. earwig (male or female) with a *Schedorhinotermes sarawakensis* termite, both showed a short antennation (less than 1.5 s) (Figure 3B,D), and the earwig then avoided the termite by moving to the left or right at an angle of up to 90° (Figure 3C). The contact never elicited an escape reaction in the earwig, and no 180° rotation of the earwig followed by back movement was recorded in adults. The behavior of the earwigs was the same in case of contact with termite workers and minor and major soldiers (Figure 3A,E). Nymphs of *S. kaja* sp. nov. showed the same pattern of avoidance with the termite, i.e., an antennation always occurred, but a rotation of 180° and moving in the opposite direction from the point of contact was recorded only in 2 of 9 observed cases. No form of mutual or unilateral aggressive behavior, grooming, mutual cleaning or other contact behavior other than antennation were observed.

When a major or minor soldier was experimentally irritated with tweezers, the soldier attacked the tweezers, opened its jaws, and expelled a drop of secretion from the frontal gland (Figure 3F). If an earwig approached such an irritated termite, the earwig responded by falling on its side in rigid immobility (thanatosis) (Figure 3F). The irritated termite never attacked the earwig, and when the soldier walked away, the earwig recovered from the short thanatosis and continued to move. After recovering from thanatosis, the earwigs showed no signs of disorientation or nonspecific convulsions, which would indicate the toxic effect of a termite secretion. This behavior was observed three times during the contact of an earwig with a major soldier; two cases of thanatosis were observed for adults (one male and one female), and one case for a nymph of 4th instar. The thanatosis lasted 28 s for an adult female, 215 s for an adult male, and 53 s for a nymph.

### 3.3. Food Composition

**Figure 4:**
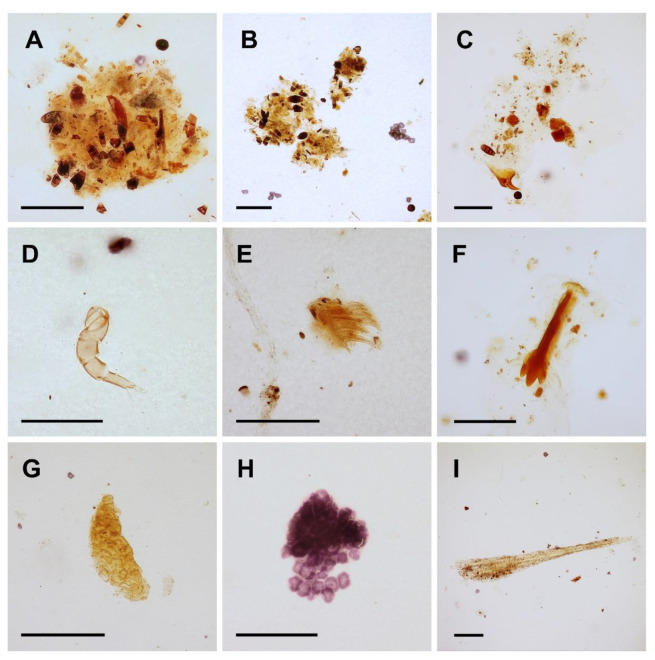
Microphotographs of gastrointestinal tract content in *Spirolabia kaja* Kočárek, sp. nov. (**A**–**C**)—chime composed of indistinguishable organic material, fungal spores, and pieces of different kinds of plant tissues; (**D**)—articulated appendage of unknown arthropod, probably a mite; (**E**,**F**)—plant trichomes; (**G**,**H**)—collenchyma tissues; (**I**)—sclerenchyma tissue. Scale bar = 0.1 mm.

The gastrointestinal tract contents of three specimens of *Spirolabia kaja* sp. nov. consisted mostly of indistinguishable organic material (detritus) (Figure 4A–I). Among the distinguishable fragments, fungal spores dominated (28 fragments), followed by the following fragments of plant tissues: indeterminate sclerenchyma tissue (1 fragment), indeterminate collenchyma tissues (9 fragments), and plant trichomes (3 fragments). Conclusive arthropod remains were represented by three fragments: one was the articulated part of an appendage (probably of a small mite), and two were indeterminate small fragments of arthropod cuticle. No fragment that demonstrably originated from the body of a host termite was documented.

## 4. Discussion

*Spirolabia kaja* sp. nov. (Spongiphoridae: Labiinae), which was found in the galleries of the wood-boring termite *Schedorhinotermes sarawakensis* (Rhinotermitidae) in Ulu Temburong National Park, Brunei Darussalam, is a termitophilic species. The occurrence of *S. kaja* sp. nov. adults together with *S. kaja* nymphs (2nd to 4th instars) in the galleries suggests that the earwig probably reproduces inside the termite colony. According to the classification of Kistner [5], *S. kaja* sp. nov. meets the parameters of a tolerated guest (a synoekete). Earwigs and termites communicated by antennation, and no forms of targeted mutual or unilateral aggressive behaviours were observed. Based on the analysis of gastrointestinal tract contents, we conclude that *S. kaja* sp. nov. is an omnivorous species that feeds on plant tissue, fungi, and occasionally on arthropod remains. The gastrointestinal tract did not contain any fragment that originated from the body of a host termite. *S. kaja* sp. nov. is therefore not a specialized predator of termites but rather a symbiont feeding on food remains prospering from the coexistence with termites for protection against predators.

*Spirolabia kaja* sp. nov. is the first earwig species for which termitophily has been demonstrated. The possible coexistence of earwigs and termites was previously indicated by the finding of the earwig *Paralabellula termitophila* (Brindle, 1970) in the nest of a nasute termite on Solomon Islands [10]. Because the latter earwig species was described based on only one male (and the species is known only from this specimen), its termitophily cannot be documented. *S. kaja* sp. nov. belongs to the same subfamily (Labiinae) as *P. termitophila*, and therefore a similar level of termitophily is probable in *P. termitophila*. The Spongiphoridae is one of two most speciose Dermaptera families, with about 500 described species [24], most of which are small and live under the bark or in the wood of dead or partly dead trees [9]. Given that they are small and occupy the same habitat, the Spongiphoridae seem to be well preadapted for association with termites, and we therefore expect that additional species in the family will be found to have associations with wood-boring termites. 

Termites are known to respond aggressively toward invaders, and species that occur with termites should therefore have adaptations that enable coexistence [25]. Conflict-avoidance behavior has been studied in inquiline species of termites, i.e., species of termites that co-occur in the nests of other termite species (e.g., [25,26,27]). For termite soldiers, aggressive behavior seems to frequently be the default response to non-nestmates [28]. With such aggressiveness in place, the appearance of any termite species other than the nest builder itself is likely to result in conflict. Inquiline species of termites reduce the chances of being attacked by reducing proximal and direct contact with the host species [26]. Hugo et al. [26] observed that, once inevitably exposed to host individuals, inquilines exhibit nonthreatening behaviors and thereby prevent conflict escalation. Such nonaggressive behavior is characterised by evasive manoeuvres that include reversing direction, bypassing hosts, and the use of defecation to repel hosts. We observed similar attack-avoidance behaviors by the termitophile earwig *S. kaja* sp. nov.; these behaviors included evasive manoeuvres and thanatosis, i.e., the feigning of death [29] (Figure 3F). The earwigs exhibited thanatosis when they were near an irritated major soldier. The trigger for this behavior was apparently the release of a drop of secretion from the soldier’s frontal gland. Because thanatosis occurred in the absence of tactile contact between the earwig and the termite soldier, we suspect that the earwig responded to an olfactory stimulus. The soldier was never observed to attack the earwig, and when the soldier walked away, the earwig began to move again. The frontal gland represents a unique adaptation of termite soldiers and is known to occur only in the advanced families Rhinotermitidae, Serritermitidae, and Termitidae [30]. The substances secreted from termite defensive glands can be used for various purposes: (1) as glues that become sticky after air exposure; (2) as contact poisons; (3) as alarm pheromones that attract soldiers and repel workers; (4) as irritants that repel, deter, or disorient; and (5) as greases that prevent wounds caused by mandibular bites from healing [31]. Kaib [32] observed the irritant properties (olfactory toxicity) of *Schedorhinotermes* vinyl ketones, which caused disorientation and cessation of recruitment by ants. The earwig thanatosis observed in the current study could be (1) a direct reaction to toxic compounds and a manifestation of temporary paralysis; (2) a defensive reaction that prevents toxin damage, because the earwig enters an inactive state possibly with closed stigmata, thereby preventing the penetration of airborne toxins into the body; or (3) a behavior that reduces the chances of being attacked by an irritated termite. Because the earwigs showed no signs of disorientation or nonspecific convulsions after recovering from the thanatosis, the earwigs did not seem to be intoxicated. We therefore suspect that the thanatosis either prevents intoxication and/or attack by an irritated termite. Cristaldo et al. [27] reported on inquiline termites that use their hosts’ alarm cues to escape danger. Our observations suggest a similar behavior in the earwig *S. kaja* sp. nov., but the reaction is not escape via movement but a reduction in the probability of attack via thanatosis. Such behavior was not previously reported for any termitophile [2,3,4,5,33,34,35] and deserves additional study. 

Unfortunately, only a small number of earwig specimens were available in the current study, and further experimental manipulations were not possible. The recorded reactions concerned only major soldiers, but a similar reaction of earwigs can be expected when *S. kaja* sp. nov. contacts irritated minor soldiers.

## 5. Conclusions

A new earwig species *Spirolabia kaja* Kočárek, sp. nov. (Spongiphoridae: Labiinae) found in an association with the wood-boring termite in a dipterocarp rain forest in Borneo is described and illustrated, and we obtained partial COI sequences from two specimens of *S. kaja* sp. nov. as DNA barcode for the purpose of molecular identification of the species. *S. kaja* sp. nov. differs from other species of *Spirolabia* in characteristic male genitalia with spirally coiled virga with 1.5 revolutions, very short parameres and medially excised apex of genital lobe. Based on behavioral observations in the laboratory, we report termite–earwig communication by antennation, and we observed no form of targeted mutual or unilateral aggressive behavior. The earwigs responded to the proximity of an experimentally irritated termite soldier by conflict-avoidance behavior based on thanatosis, which seems to be a defensive reaction that may reduce the chance of being attacked by an irritated termite. To assess the possibility of predation on termites, we analysed gastrointestinal tract contents, and we conclude that *S. kaja* sp. nov. is an omnivorous species that feeds mainly on plant tissues and fungi and only occasionally on arthropod remains. The occurrence of *S. kaja* sp. nov. adults together with the nymphs (2nd to 4th instars) in the galleries of *S. sarawakensis* suggests that the earwig can reproduce inside the termite colony. *Spirolabia kaja* Kočárek, sp. nov. is the first earwig species for which termitophily has been demonstrated.

## Data Availability

The holotype of *Spirolabia kaja* sp. nov. is deposited in the collection the National Museum, Praha, Czech Republic (NMPC); in the paratypes in NMPC, in the Institute for Biodiversity and Environmental Research, Universiti Brunei Darussalam, Brunei Darussalam (UBDC); and in the collection of P. Kočárek, University of Ostrava, Czech Republic (PKCO).

## References

[1] Fewel J., Abbot P., Córdoba-Aguilar A., González-Tokman D., González-Santoyo I. (2018). Sociality. Insect Behavior: From Mechanisms to Ecological and Evolutionary Consequences.

[2] Parmentier T., Starr C. (2020). Guests of social insects. Encyclopedia of Social Insects.

[3] Korb J., Bignell D., Roisin Y., Lo N. (2010). Termite Mound Architecture, from Function to Construction. Biology of Termites: A Modern Synthesis.

[4] Wilson E.O. (1971). The Insect Societies.

[5] Kistner D.H., Herman H.R. (1979). Social and evolutionary significance of social insect symbionts. Social Insects.

[6] Kistner D.H. (1990). The integration of foreign insects into termite societies or why do termites tolerate foreign insects in their societies?. Sociobiology.

[7] Howard R.W., McDaniel C.A., Blomquist G.J. (1980). Chemical Mimicry as an Integrating Mechanism: Cuticular Hydrocarbons of a Termitophile and Its Host. Science.

[8] Costa D.A., de Carvalho R.A., de Lima Filho G.F., Brandao D. (2009). Inquilines and invertebrate fauna associated with termite nests of Cornitermes cumulans (Isoptera, Termitidae) in the Emas National Park, Mineiros, Goiás, Brazil. Sociobiology.

[9] Haas F., Foottit G.R., Adler P.H. (2018). Biodiversity of Dermaptera. Insect Biodiversity: Science and Society.

[10] Brindle A. (1970). The Dermaptera of the Solomon Islands. Pac. Insects.

[11] Steinmann H. (1990). Dermaptera: Eudermaptera I. Tierreich 106.

[12] Sakai S. (1993). Dermapterorum Catalogus XXV: Iconographia IX. Explicatio Series VIIIb: Spongiphoridae Verhoeff (1902) et Kevan (1980). III.

[13] Maiti P.K. (2006). A taxonomic monograph on the world species of termites of the family Rhinotermitidae (Isoptera: Insecta). Mem. Zool. Surv. India.

[14] Kamimura Y. (2013). Pre- and postcopulatory sexual selection and the evolution of sexually dimorphic traits in earwigs (Dermaptera). Èntomol. Sci..

[15] Kocarek P., Dvorak L., Kirstova M. (2015). Euborellia annulipes (Dermaptera: Anisolabididae), a new alien earwig in Central Eu-ropean greenhouses: Potential pest or beneficial inhabitant?. Appl. Entomol. Zool..

[16] Anderson L.E. (1954). Hoyer’s solution as a rapid permanent mounting medium for bryophytes. Bryologist.

[17] Folmer O., Black M., Hoeh W., Lutz R., Vrijenhoek R. (1994). DNA primers for amplification of mitochondrial cytochrome c oxidase subunit I from diverse metazoan invertebrates. Mol. Mar. Biol. Biotechnol..

[18] Srivastava G.K. (1976). Catalogue of Oriental Dermaptera. Rec. Zool. Surv. India Occas. Pap..

[19] Srivastava G.K. (1995). On the classification of *Spongiphoridae (=Labiidae)* with a list of Dermaptera. Rec. Zool. Surv. India.

[20] Steinmann H. (1989). World catalogue of Dermaptera. Ser. Entomol..

[21] Steinmann H. (1987). Two new genera and species for the subfamily *Labiidae (Dermaptera: Labiidae)*. Acta. Zool. Hung..

[22] Kevan D.K.M.E., Vickery V.R. (1997). An annotated provisional list of non-saltatorial orthopteroid insects of Micronesia, compiled mainly from the literature. Micronesia.

[23] Kamimura Y., Nishikawa M., Lee C.-Y. (2016). The earwig fauna (Insecta: *Dermaptera*) of Penang Island, Malaysia, with descriptions of two new species. Zootaxa.

[24] Hopkins H., Maehr M.D., Haas F., Deem L.S. Dermaptera Species File. http://Dermaptera.SpeciesFile.org.

[25] Shellman-Reeve J., Choe J.C., Crespi B.C., Fraser S. (1997). The spectrum of eusociality in termites. The Evolution of Social Behaviour in Insects and Arachnids.

[26] Hugo H., Cristaldo P.F., DeSouza O. (2020). Nonaggressive behavior: A strategy employed by an obligate nest invader to avoid conflict with its host species. Ecol. Evol..

[27] Cristaldo P.F., Rodrigues V.B., Elliot S.L., Araújo A.P., DeSouza O. (2016). Heterospecific detection of host alarm cues by an in-quiline termite species (Blattodea: Isoptera: Termitidae). Anim. Behav..

[28] Noirot C., Krishna K., Weesner F.M. (1970). The nests of termites. Biology of Termites.

[29] Sherratt T.N., Kang C., Córdoba-Aguilar A., González-Tokman D., González-Santoyo I. (2018). Anti-predator behavior. Insect Behavior: From Mechanisms to Ecological and Evolutionary Consequences.

[30] Prestwich G.D. (1984). Defense mechanisms of termites. Annu. Rev. Entomol..

[31] Šobotník J., Bourguignon T., Hanus R., Weyda F., Roisin Y. (2010). Structure and function of defensive glands in soldiers of Glos-sotermes oculatus (Isoptera: Serritermitidae). Biol. J. Linn. Soc..

[32] Kaib M., Breed M.D., Michener C.D., Evans H.E. (2019). Disruption of Ant Recruitment by the Frontal Gland Secretion of a Termite: A Chemical Defense Strategy. The Biology of Social Insects.

[33] Matthews R.W., Matthews J.R. (2010). Insect Behavior.

[34] Bignell D.E., Roisin Y., Lo N. (2011). Biology of Termites: A Modern Synthesis.

[35] Abe T., Bignell D.E., Higashi M., Higashi T. (2000). Termites: Evolution, Sociality, Symbioses, Ecology.

